# Insight into the Way the Content of Biologically Active Compounds in Meadowsweet Inflorescences (*Filipendula ulmaria* (L.) Maxim.) Is Shaped by Phytosociological Habitats

**DOI:** 10.3390/molecules26175172

**Published:** 2021-08-26

**Authors:** Kinga Stawarczyk, Aleksandra Chrupek, Agnieszka Sękara, Michał Gostkowski, Małgorzata Karbarz

**Affiliations:** 1 Department of Biology, Institute of Biology and Biotechnology, University of Rzeszow, Pigonia Street 1, 35-310 Rzeszów, Poland; kstawarczyk2@o2.pl; 2 Department of Horticulture, Faculty of Biotechnology and Horticulture, University of Agriculture in Krakow, 29 Listopada 54, 31-425 Krakow, Poland; chrupek92@gmail.com (A.C.); a.sekara@ur.krakow.pl (A.S.); 3 Department of Econometrics and Statistics, Institute of Economics and Finance, Warsaw University of Life Sciences-SGGW, 02-776 Warsaw, Poland; michal_gostkowski@sggw.edu.pl

**Keywords:** meadowsweet, phenolics, salicylates

## Abstract

A collection of herbs from the natural environment remains not only a source of raw material but also provides evidence of chemical differentiation of the local populations. This work aimed at performing a phytosociological analysis of seven different stands of meadowsweet (*Filipendula ulmaria* (L.) Maxim.) occurrence. A determination of total phenolic compounds and salicylates and the antioxidant activity of dried meadowsweet inflorescences (*Flos ulmariae*) was also performed. Active chemical compounds in *F. ulmaria* inflorescences were related to chemotype and diversified between investigated populations. Geographical distance and variation in phytosociological locations affected chemical composition in different ways, shaping the content of biochemical compounds crucial for herbal material quality. The obtained results can be a valuable indicator for Nexo and Baligród populations, which are good genetic material for research, breeding, and cultivation due to their biochemical composition, especially with respect to salicylates, as major compounds of determining market quality of *Flos ulmariae*.

## 1. Introduction

Meadowsweet (*Filipendula ulmaria* (L.) Maxim., Syn. *Spiraea ulmaria* L., *Rosaceae*) is a herbaceous perennial, flowering from May to August, associated with moist or wet habitats such as marshes, peat bogs, ditches, banks, wet deciduous forests, wet meadows, and mountain herbal communities as well as northern grassy layers, which are often dry. This species occurs on neutral or limestone soils. Meadowsweet is native to Asia (Mongolia, Siberia, China) and northern, central, and eastern Europe, except for the Arctic and the Mediterranean, while the cultivation area covers Europe and North America [1,2,3,4]. Meadowsweet is a plant 80–100 cm tall, with a short, horizontal rhizome. The leaf blades are pinnate, with 2–5 pairs of leaflets. Inflorescence branches and pedicels are glabrous or tomentose, composed of more than 100 flowers. Flowers consist of green sepals and white to cream petals, 7–8 mm in diameter. The fruit is dry, 0.3–0.4 mm in length [5].

In folk medicine, all parts of the plant (flowers, leaves, roots) have been used in the treatment of many diseases, in the form of stocks, water extracts, tinctures, and ointments. Flowers of the meadowsweet were applied in the treatment of rheumatism, gout, cold, fever, infections, and peptic ulcer disease because of their anti-inflammatory, analgesic, antibacterial, antioxidant, and anti-cancer activity [4,6,7,8]. The European Medicines Agency approved the use of meadowsweet flowers, *Filipendulae ulmariae flos*, for the preparation of traditional herbal medicinal products [6]. Meadowsweet flowers are officially recommended for use in medicine as an anti-inflammatory, wound-healing, and astringent remedy [9].

The healing properties of the meadowsweet are mainly due to specific polyphenolics of an anti-inflammatory, antioxidant, and antimicrobial nature (e.g., total phenols, salicylates, quercetin). The salicylic acid component is released via oxidation from its aglycones (e.g., salicylaldehyde, methylsalicylate) developed from glycosides through hydrolysis in the human digestive system [10,11,12]. The raw material is rich in flavonoids (spiraeoside, rutoside, quercetin-3-glucuronide, hyperoside, spiraeoside, quercetin 4′-glycoside), phenolic glycosides, tannins, and essential oils (methyl salicylate, vanillin, salicylaldehyde, heliotropin). The flowers also contain organic acids (in addition to salicylic, citrate, and ascorbic acids), coumarins, isosalicin, and monotropitoside (gaultherin) [13]. We hypothesized that active chemical compound content in *F. ulmaria* flowers is related to the chemotype and diversified between population habitat type, biological community, physiographic features, or other natural characteristics. Geographical distance and variation in phytosociological locations should affect the chemical composition in different ways. The aim of this work was to perform a phytosociological analysis of seven different populations of the meadowsweet (*Filipendula ulmaria* (L.) Maxim.). A determination of total phenolic compounds and salicylates was also performed, and the antioxidant activity of dried meadowsweet flowers (*Flos ulmariae*) was collected from chosen localities to point to those most promising for future investigations.

## 2. Results

The floristic composition of the researched locations (Table 1) allowed us to classify them to the compound *Filipendulion ulmariae*, which partly refers to natural shrub communities composed of high dicotyledon perennials occurring along watercourses on mainly organic fertile and medium fertile habitats. Meadowsweet was the dominant species in these communities. In the original vegetation, delta communities of forest and shrub formation were most common and later evolved into wet hay meadows in the order of *Molinietalia* [14]. Soil fertility on the surveyed locations confirmed the high stability (III) of occurrence of the nettle (*Urtica dioica*), which prefers nitrogen-rich soils. Due to the occurrence of the Livaria (*Lythrum salicaria*) genus in Baligród and Iskrzynia, these locations can be included in the *Lythro-Filipendulateum ulmariae* group. Bornholm, Allinge-Sandvig is an atypical location. Due to very variable environmental conditions—dry rocks and humid depressions—it includes species characteristic both for dry habitats, e.g., white swallow-wort (*Vincetoxicum hirundinaria*) and rock garlic (*Allium montanum*), and humid ones, e.g., meadowsweet (*Filipendula ulmarie*) and lotora (*Lotus uliginosus*).

**Figure 1:**
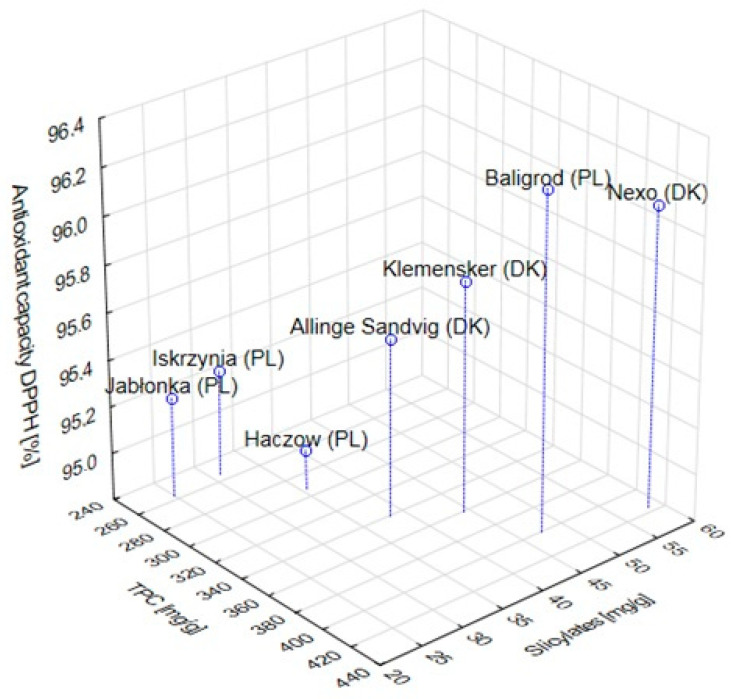
Location of analyzed locations in three-dimensional space.

Chemical parameters of meadowsweet inflorescences are presented in Table 2. The tested herbal raw material showed a positive correlation between all examined traits. The highest value of correlation (r = 0.89) was determined between the content of salicylates and total polyphenols, which proved that salicylates are the main phenolic compounds occurring in the meadowsweet. This fact is confirmed by a high correlation coefficient between salicylate content and antioxidant activity (r = 0.73). A high correlation was also determined between the polyphenols content and antioxidant activity of the examined plant material (r = 0.80). The reason is that polyphenols are the main substances responsible for antioxidant properties. Derogations may be due to the occurrence of other substances exhibiting antiradical activity in the meadowsweet, e.g., alpha-tocopherol, ascorbic acid, or carotenoids. The correlation coefficients analysis showed a significant connection between all the studied parameters characterizing the quality of herbal raw material from all locations (Figure 1).

**Figure 2:**
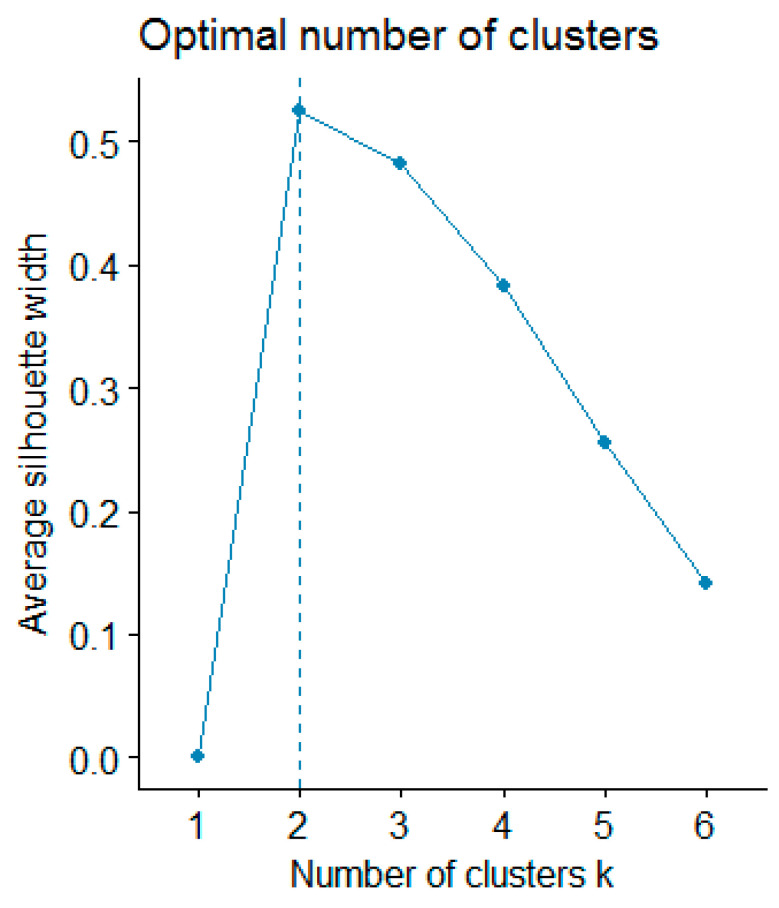
Value of average silhouette index for the different number of clusters.

In the next step, hierarchical clustering based on obtained results was performed to group objects according to their similarity. To achieve this goal, agglomerative clustering with Euclidean distance and Ward’s linkage method was used. The data were standardized before clustering. The average silhouette index was used to determine the optimal number of clusters (Figure 2).

Higher value indicates better clustering and the highest value should be considered as the optimal number of clusters. Source: own preparation.

**Figure 3:**
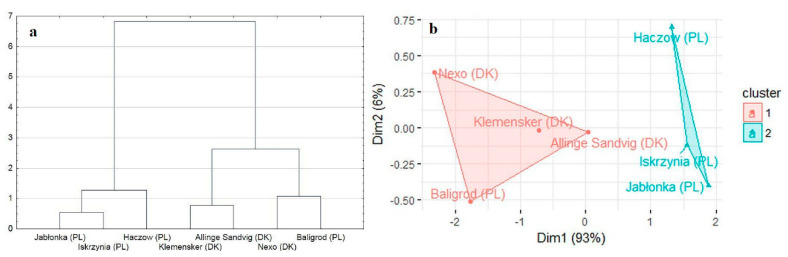
(**a**) Dendrograms showing the clustering algorithm after the utilization of principal component analysis; (**b**) Graph presenting final clusters after the utilization of principal component analysis.

The result of clustering is presented in Figure 3. The performed analysis showed that the analyzed objects could be divided into two groups. The first group contains Jabłonka (PL), Haczów (PL), and Iskrzynia (PL). The second group contains Baligrod (PL), Allinge-Sandvig (DK), Klemensker (DK), and Nexo (DK).

**Figure 4:**
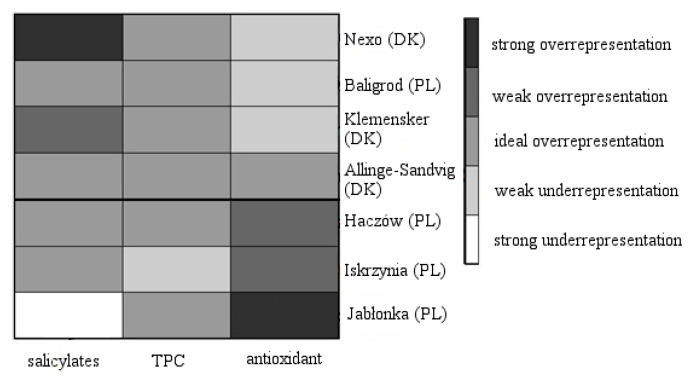
Overrepresentation maps for analyzed objects. Strong (under) overrepresentation indicates (lower) higher value in relation to mean value of analyzed variable. Source: own preparation.

Additionally, GCA analysis was performed (Figure 4). The overrepresentation maps showed that objects with a higher level of antioxidant capacity were selected in the first cluster. On the other hand, the second cluster contained objects with a lower level of antioxidant capacity and a higher level of salicylates. 

## 3. Discussion

About two-thirds of the 50,000 different species of medicinal plants used are obtained from the natural environment. Only 10% of commercial medicinal species are grown in Europe [15]. For this reason, it is necessary to investigate factors that may affect the quality of the raw material collected from natural habitats, including the content of active substances. Most herbal plants contain substances whose biological activity is associated with their antioxidant activity. These include mainly polyphenols but also carotenoids, saponins, vitamins (ascorbic acid, vitamin E), and micronutrients (zinc, selenium, copper). The reducing abilities of these compounds consist of connecting the missing electron to free radicals. As a result of this reaction, the free radicals undergo oxidization, and are consequently characterized by very low reactivity [9,11,12]. Harboure et al. [10] determined the content of phenols in organic meadowsweet flowers grown in the city of Stokestown in Ireland. Depending on the method of drying, the phenols content in the tested raw material ranged from 110 to 119 mg g^−1^ of dry weight [10]. This is over two times less than in the natural sites studied, which indicates the adverse effect of growing conditions in the cited experiments on the phenolic content in the raw material of this species.

Fecka et al. [2] analyzed the polyphenols content in meadowsweet flowers from different companies—Kawon, Flos, Herbapol, and Herbalux—operating on the Polish market [2]. In the cited study, the average polyphenols value was 131.65 mg g^–1^ of dry weight, at least two times lower than those obtained in the present research on meadowsweet collected from natural locations. Such differences may result from the fact that herbs authorized for sale must undergo a decontamination process, which leads to partial degradation of biologically active ingredients. Vysochina et al. [16] determined the content of phenols in meadowsweet growing naturally in the Ural mountains. The results obtained were in the range of 83–129 mg g^−1^ of dry weight, i.e., at least 50% less than in the positions we examined [16]. This indicates the adverse impact of high mountain conditions on the synthesis of phenols. Correlation analysis showed that the polyphenol content was closely related to salicylates and antioxidant capacity, which is evidence that the main phenolic compounds contained in the meadowsweet are salicylates.

In terms of popularity, the meadowsweet is the second salicylic material used in folk medicine and modern phytotherapy [10] after the willow (*Salix* L.). According to Harbourne et al. (2009), compared to the willow, this species contains fewer salicylates, while the total polyphenol content is twice as high [17]. Study of Olennikov [13] provided detailed data on the nutritional profile, phenolic, essential oil, water-soluble polysaccharide composition, and bioactivity of herbal products used in Siberia, namely *Fulmaria* floral teas. As far as we know, this is the first study reporting data on these parameters for the mentioned products. Macronutrients were found in appropriate amounts in meadowsweet teas, with carbohydrates being the predominant components. Phenolic profiling of the meadowsweet teas revealed high contents of flavonols and ellagitannins. Interestingly, the meadowsweet teas were also found to be a source of bioactive volatiles such as salicylaldehyde and methyl salicylate, which are the components of the essential oils of *Filipendula* flowers. It should be noted that the water-soluble components were characterized by the presence of polymeric carbohydrates with a high content of galactose. The bioactivity data demonstrated the good ability of meadowsweet teas to inhibit amylase, glucosidase and AGE formation, and also expressed antioxidant properties [13]. 

Based on the obtained results, two groups of local populations can be distinguished regarding the main biologically active components of the meadowsweet inflorescences. Chemical parameters such as DPPH and TPC depend not only on genetic variability but also on environmental variability, for example, climate and soil conditions. 

The Bieszczady climate is mountainous with relatively strong continental features. The average annual temperature fluctuates in the range of 4–5 °C to 7–7.5 °C in the Bieszczady Foreland. The average temperature is 14–15 °C in summer, and −3 °C in winter (up to −7 °C in the highest parts of the mountains) [18]. Meanwhile, Bornholm has a moderate maritime climate slightly different from the rest of Denmark, mainly due to the influence of the Baltic Sea. In general, the climate here is milder than in the rest of Denmark. Bornholm gets the highest amount of hours of annual sunshine in Denmark. However, during the winter, longer periods with subzero temperatures (during the night) are common. The Baltic Sea freezes over during some winters [19]. When this occurs, the warming effect of the sea disappears and the temperature drops. The results of the investigated parameters could also be influenced by the proximity of other plants (Table 1) and their allelopathic interactions, pathogens, and pests. 

Kornaś and Miedwiecka-Kornaś [20] determined that the abovementioned factors are crucial for the co-evolution and diversification of plant communities as well as the biochemical and physiological status of individuals within communities [20]. Our research showed that the polyphenols content in the meadowsweet was variable and dependent on the location from which the plant material was collected. Differences in the content of phenols in the studied populations may have resulted from different soil conditions, competitive and/or antagonistic interactions between plants, and the variability of environmental conditions during plant ontogeny. Because phenolic compounds are synthesized in a plant’s response to stress, it is important for stress conditions at a given position as well as the frequency of stressors to be taken into account [11,12]. A stressful environment can force plants to biosynthesize more phenolic compounds in comparison to plants growing under optimal conditions. Phenolics are capable of scavenging free radicals that result in cell membrane peroxidation’s reduction. This is an antioxidative role that protects plant cells from oxidative stress. Under stressful conditions, phenolics biosynthesis is regulated by key enzymes with altered activities. These enzymes, such as PAL (phenylalanine ammonia-lyase) and CHS (chalcone synthase), are crucial in phenolic biosynthetic pathways. The transcript level of genes encoding key biosynthetic enzymes such as PAL, C4H (cinnamate 4-hydroxylase), 4CL (4-coumarate: CoA ligase), CHS, CHI (chalcone isomerase), F3H (flavanone3-hydroxylase), F30H (flavonoid 30-hydroxylase), F3050H (flavonoid 3050-hydroxylase), DFR (dihydroflavonol 4-reductase), FLS (flavonol synthase), IFS (isoflavone synthase), IFR (isoflavone reductase), and UFGT (UDP flavonoid glycosyltransferase) is up-regulated. Other abiotic factors such as temperature, nanoparticles, and pesticides also stimulate endogenous phenolic biosynthesis in plants and help to provide resistance against the phytotoxic effects of these abiotic stresses [21]. The highest level of phenolic compounds was marked in the inflorescences of the meadowsweet collected in Nexo (DK), Baligród (PL) and Klemensker (PL). 

Moreover, the plant composition of these sites (Nexo–DK, Baligród–PL and Klemensker–DK) included yarrow (*Achillea millefolium*)—a species typical for soils with high humus content, which contains phenolic compounds. Prominent theories of plant defense have predicted that plants growing on nutrient-poor soils produce more phenolic defense compounds than those on richer soils. Nitrogen and phosphorus limitations could have different effects, because these nutrients are involved in different cellular metabolic processes. N limitation reduces protein production and thus competition for phenylalanine, a precursor of many phenolic compounds. In contrast, P acts as a recyclable cofactor in these reactions, allowing protein and hence phenolic production to continue under low P conditions [22]. The smallest number of phenolic compounds was determined at the sites of Haczów, Iskrzynia, and Jabłonka. A common feature of these sites belonging to a single cluster is the presence of the indicator species for soils rich in nitrogen: the nettle (*Urtica dioica* L). This may indicate that soils with high nitrogen content are unfavorable to the meadowsweet in the aspect of synthesis of phenolic compounds, or that the meadowsweet demonstrates high competitiveness with the nettle in terms of nutrient uptake from the soil. 

## 4. Materials and Methods

**Figure 5:**
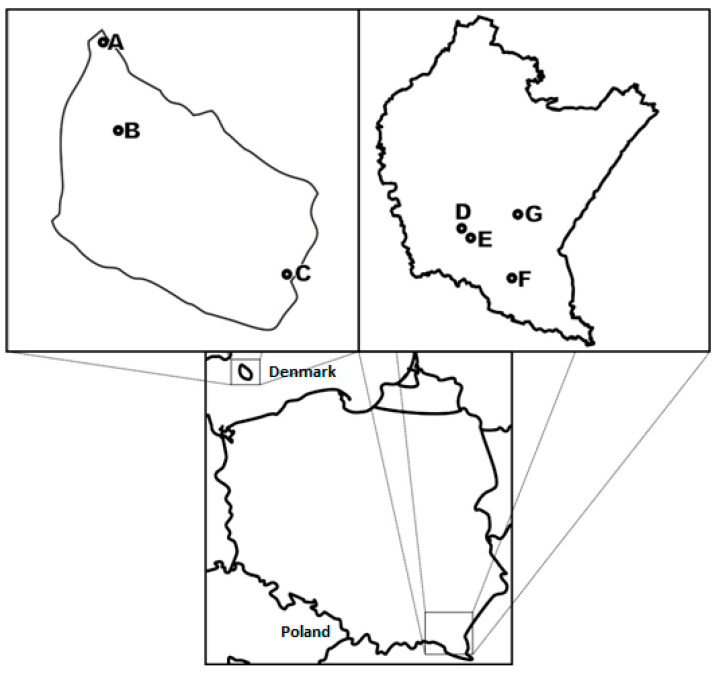
Individual locations of the meadowsweet. Denmark: A: Alinge-Sandvig (55°16′24.00″ N, 14°48′2.99″ E), B: Klemensker (55°10′27″ N 14°48′24″ E), C: Nexo (55°04′ N 15°09′ E), Poland: D: Iskrzynia (49°41′01″ N 21°51′21″ E), E: Haczów (49°40′09″ N 21°53′28″ E), F: Baligród (49°20′14″ N, 22°17′09″ E), G: Jabłonka (49°41′48″N 22°07′01″E), Source: own preparation.

**Figure 6:**
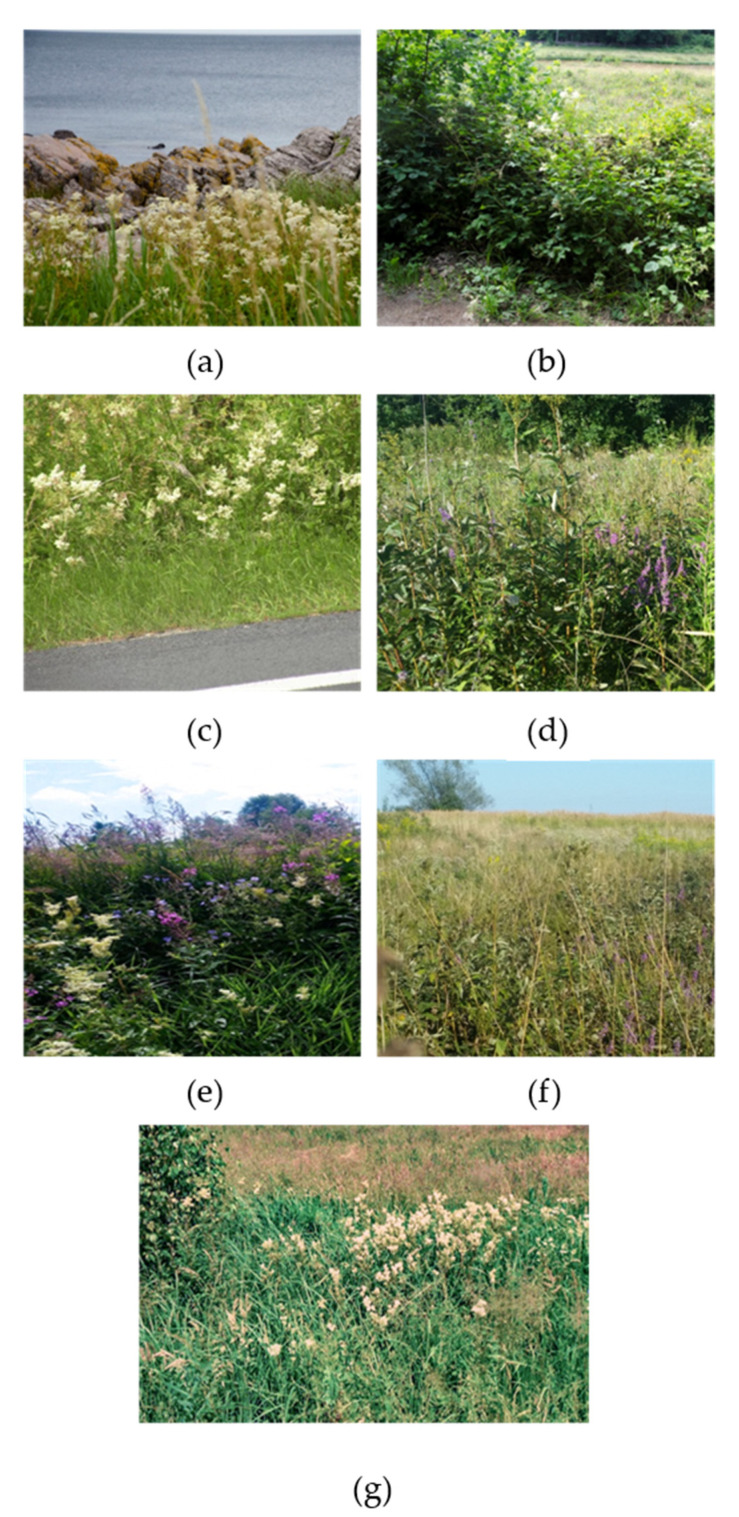
Individual locations of the meadowsweet: Alinge-Sandvig (**a**), Klemensker (**b**), Nexo (**c**), Iskrzynia (**d**), Haczów (**e**), Baligród (**f**), Jabłonka (**g**).

The investigations were performed in the meadowsweet’s natural areas of occurrence (*Filipendula ulmaria* (L.) Maxim.). Two main locations (Poland, PL, and Denmark, DK) were chosen on the basis of geographical distance, within which specific locations—Baligród (PL), Haczów (PL), Iskrzynia (PL), Jabłonka (PL), Allinge-Sandvig (DK), Klemensker (DK), and Nexo (DK)—were selected on the basis of phytosociological diversity. At each locality, the plant species were identified by at least two taxonomists representing the author’s team. The floristic composition of *Filipendula ulmaria* locations was presented in Table 1, individual locations were documented within Figure 5 and Figure 6. 

### 4.1. Phytosociological Analysis

The phytosociological image areas, of a surface of 100 m^2^ each, were selected on the basis of a biased test, i.e., the documentation was subjected to plant communities that were characterized by meadowsweet occurrence. The criterion of botanical richness focused on high-quality species assemblages (targeting areas for richness assessment) as well as the purposive sampling technique (areas with potential for sustainable meadowsweet harvesting) were applied in the selection of the sites [23,24]. Basing on mentioned criteria and authors, the locations in Poland and Denmark were chosen as examples of different ecological conditions. The locations were described using the Global Positioning System (GPS), Android Application Package. The characteristic species of syntaxonomic units and the system of distinguished sites were given according to the Matuszkiewicz classification, where species incidence were marked with degree of abundance (+, 1, 2, 3, 4, 5) and stability of the species was defined as the relative frequency of the species within the syntaxone. It is expressed in the percentage of phytocoenoses in which a given species occurs in relation to the total number of phytocoenoses included in a given syntaxon [25].

### 4.2. Plant Material Collection

About 500 g of fresh inflorescences were collected from each stand during the phase of full flowering, according to WHO guidelines on good agricultural and collection practices (GACP) for medicinal plants [26]. The material was collected in mid-July, in the afternoon, during sunny weather, and samples were then subjected to natural drying. Drying took place in an airy and shady attic; the inflorescences were tied in bunches and attached to the ceiling.

### 4.3. Chemical Analysis

Total antioxidant activity was determined according to the 2,2-diphenyl-1-picrylhydrazyl (DPPH) radical reduction method [27,28]. The dried samples were ground and homogenized, 2.5 g of dried sample was mixed with 10 mL of 80% methanol and centrifuged (3492× *g*) for 10 min at 4 °C. The decanted supernatant in a volume of 0.1 mL and 0.1 mM DPPH was dissolved with 4.9 mL of 80% methanol. The mixture was incubated for 15 min, in the dark, at the temperature of 20 °C. The absorbance was measured at 517 nm using a UV–Vis Helios Beta spectrophotometer (Thermo Fisher Scientific Inc., Waltham, WA, USA). DPPH∙ radical scavenging activity was calculated with the formula: AA [%] = [(A_0_ − A_1_)/A_0_] × 100; where AA was the antioxidant activity, A_0_—the absorbance of the reference solution, and A_1_—the absorbance of the test solution.

Salicylates were determined by the colorimetric method using FeCl_3_ according to Warrier et al. [29]. The sample weight of 0.1 g of dry vegetable material was triturated with 10 mL of hot 0.5 M NaOH, filtered through a soft filter into a 100 mL graduated flask, and made up to volume with distilled water. The test tubes contained 2 mL of filtrate and 8 mL of 0.02 M FeCl_3_. The absorbance was measured at 530 nm [29]. 

Total phenolics were estimated using the modified Folin–Ciocalteu colorimetric method [30]. Plant material (2.5 g) was mixed with 10 mL 80% methanol. Samples were subjected to centrifugation (15 min, 3492× *g*, 4 °C). The glass tubes contained 0.1 mL of supernatant and 2 mL sodium carbonate. After the next 5 min, 0.1 mL Folin–Ciocalteu’s reagent, mixed with deionized water (1:1 *v/v*), was added to the test tubes. The standard curve was prepared using gallic acid dilutions. The absorbance of the mixture was measured at 750 nm against a reference solution. The results are expressed as mg gallic acid equivalents (GAE) per 1 g fresh weight.

### 4.4. Statistical Analysis

To determine the differences between the average measurement results of salicylate content, phenol compound content, and antioxidant activity, a one-way analysis of variance (factor: location) and Tukey’s HSD test were performed. To examine the correlation with antioxidant strength values, phenolic compounds content, and salicylate content in the tested plant material, Pearson correlation coefficients (r) were calculated. 

In the next step, hierarchical clustering was performed to group objects based on their similarity. To achieve this goal, agglomerative clustering with Euclidean distance and Ward’s linkage method were used. The data were standardized before clustering. The average silhouette index was used to determine the optimal number of clusters.

Grade Correspondence Analysis (GCA) was also a method used in these studies. An important feature of GCA is the fact that it does not build a new synthetic measure but takes into account the original structure of the phenomenon. The overrepresentation index indicates the extent to which the observed value differs from that which would be expected from ideal proportionality distribution (i.e., when there exists no relationship between rows and columns). For such a set of overrepresentation indicators, a map showing a degree of data representation can be created. With a few shades of gray areas of underrepresentation, the ideal representation and overrepresentation of the data can be identified. In the paper, the areas are identified as follows: a value below 0.8 indicates strong underrepresentation, a value in the range of 0.8–0.98 determines there is poor underrepresentation, a value in the range of 0.98–1.02 is an ideal representation [31].

## 5. Conclusions

The collection of herbs from the natural environment remains not only a source of raw material but also provides evidence of chemical differentiation of the local populations. We demonstrated that meadowsweet inflorescences collected from seven natural areas in Poland and Denmark are a valuable source of compounds determining its pharmacological activity. The variation in the content of total phenolics, including salicylates, may result from the geographical distance between the locations in which samples were collected (PL and DK) as well as from ecological characteristics of a given area, which shaped plant populations with different chemotypic inheritance. Additionally, the obtained results can be a valuable indicator for populations occurring on the island of Bornholm, as well as those collected from the area of Baligród, which are a good genetic material for research, breeding, and cultivation due to their biochemical composition, especially with respect to salicylates, as major compounds of determining market quality of *Flos ulmariae*.

## Figures and Tables

**Table 1:** Phytosociological analysis of meadowsweet locations.

Location	Allinge-Sandvig (DK)	Klemensker (DK)	Nexo (DK)	Iskrzynia (PL)	Haczów (PL)	Baligród (PL)	Jabłonka (PL)	Stability
Number of species in the photo ^1^	24	9	16	14	11	21	12	
ChCl. *Molinio-Arrhenatheretea*								
*Festuca pratensis*	- ^2^	1	-	1	-	-	-	II ^3^
*Rumex acetosa*	-	-	-	-	+	-	-	I
*Rannunculus acris*	-	-	-	-	+	1	-	I
*Vicia cracca*	-	-	-	-	-	-	-	I
ChO. *Molinietalia caerulae*								
*Crisum palustre*	-	-	-	-	-	-	1	I
*Lotus ulignosus*	-	1	-	-	-	-	-	I
*Eupatorium cannabinum*	-	-	-	-	-	-	1	I
ChAll. *Filipendulion ulmariae*								
*Filipendula ulmaria*	4	2	3	3	1	4	2	V
*Hypericum tetrapterum*	-	-	-	-	-	-	2	I
*Lythrum salicaria*	-	-	-	2	-	1	-	II
*Euphorbia palustris*	-	-	-	-	-	1	-	I
*Mentha longifolia*	-	-	-	-	-	+	-	I
*Stachys palustris*	-	-	+	-	-	-	-	I
ChAll. *Caltion palustris*								
*Juncus effuses*	-	-	-	-	-	2	-	I
*Lysimachia vulgaris*	-	-	-	-	-	1	-	I
ChO. *Arrhenatheretalia*								
*Dactylis glomerata*	-	-	-	+	-	-	-	I
*Equisetum palustre*	-	-	-	-	+	-	-	I
*Campanula patula*	-	-	-	+	-	+	-	II
*Crepis biennis*	-	-	-	-	1	2	-	II
*Geranium pretense*	-	-	-	-	1	+	-	II
*Achillea millefolium*	-	-	-	+	+	1	-	III
*Arrhenantheum eliatus*	-	1	-	-	-	-	-	I
ChO. *Agrorypo-Rumicon crispi*								
*Festuca arundinacea*	-	-	-	1	1	-	-	II
Associated species								
ChCl. *Artemisietea vulgaris*								
*Artemisia vulgaris*	+	-	-	-	-	-	-	I
*Solidago canadiensis*	-	-	-	2	-	+	-	II
*Urtica dioica*	-	-	-	1	+	-	+	III

^1^ photo’s area of 100 m^2^, ^2^ degree of abundance (+, 1, 2, 3, 4, 5); - lack of species, ^3^ I—1–20%, II—21–40%, III—41–60%, IV—61–80%, V—81–100% of occurrences.

**Table 2:** Chemical parameters of meadowsweet inflorescences.

Location	Total Phenolics (mg g^−1^ DW)			Slicylates (mg g^−1^ DW)			Antioxidant Capacity (%DPPH)		
	X_max_–X_min_	Mean	σ	X_max_–X_min_	Mean	σ	X_max_–X_min_	Mean	σ
Allinge-Sandvig (DK)	344.64–336.48	341.47 ^b^*	4.37	40.23–35.49	38.23 ^bcd^	2.45	95.67–95.45	95.56 ^b^	0.11
Klemensker (DK)	385.44–336.48	360.96 ^b^	24.48	45.41–42.39	44.26 ^cd^	1.65	96.00–95.56	95.78 ^b^	0.22
Nexo (DK)	438.48–404.48	417.17 ^c^	18.56	63.52–54.03	58.34 ^e^	4.8	96.34–95.78	96.08 ^c^	0.28
Iskrzynia (PL)	260.32–248.08	255.33 ^a^	6.43	34.2–28.6	30.9 ^ab^	2.93	95.56–95.01	95.26 ^a^	0.28
Haczów (PL)	309.28–282.08	293.87 ^a^	13.96	41.53–28.17	35.5 ^abc^	6.77	95.23–94.67	94.97 ^a^	0.28
Baligród (PL)	412.64–390.88	400.40 ^c^	11.13	51.01–43.86	47.28 ^de^	3.67	96.45–95.89	96.23 ^c^	0.29
Jabłonka (PL)	264.40–249.44	257.15 ^a^	7.49	29.46–21.7	24.87 ^a^	4.07	95.45–94.89	95.23 ^a^	0.29

*^a–e^ Different letters in the same column indicate significant statistical difference (*p* < 0.05, Tukey’s HSD test). Data are means of three replicates.

## Data Availability

Most data supporting the results are included in the article. The datasets used and/or analyzed during the current study are available from the corresponding author on reasonable request.

## Sample Availability

Samples of the compounds are not available from the authors.
